# Modelling COVID-19 vaccine breakthrough infections in highly vaccinated Israel—The effects of waning immunity and third vaccination dose

**DOI:** 10.1371/journal.pgph.0001211

**Published:** 2022-11-09

**Authors:** Anyin Feng, Uri Obolski, Lewi Stone, Daihai He

**Affiliations:** 1 Department of Applied Mathematics, The Hong Kong Polytechnic University, Hong Kong, China; 2 School of Public Health, Faculty of Medicine, Tel Aviv University, Tel Aviv, Israel; 3 Porter School of the Environment and Earth Sciences, Faculty of Exact Sciences, Tel Aviv University, Tel Aviv, Israel; 4 Mathematical Sciences, School of Science, RMIT University, Melbourne, Australia; 5 Biomathematics Unit, School of Zoology, Faculty of Life Sciences, Tel Aviv University, Tel Aviv, Israel; 6 Research Institute for Future Food, The Hong Kong Polytechnic University, Hong Kong, China; The University of Sydney, AUSTRALIA

## Abstract

In August 2021, a major wave of the SARS-CoV-2 Delta variant erupted in the highly vaccinated population of Israel. The transmission advantage of the Delta variant enabled it to replace the Alpha variant in approximately two months. The outbreak led to an unexpectedly large proportion of breakthrough infections (BTI)–a phenomenon that received worldwide attention. Most of the Israeli population, especially those aged 60+, received their second dose of the vaccination four months before the invasion of the Delta variant. Hence, either the vaccine induced immunity dropped significantly or the Delta variant possesses immunity escaping abilities, or both. In this work, we model data obtained from the Israeli Ministry of Health, to help understand the epidemiological factors involved in the outbreak. We propose a mathematical model that captures a multitude of factors, including age structure, the time varying vaccine efficacy, time varying transmission rate, BTIs, reduced susceptibility and infectivity of vaccinated individuals, protection duration of the vaccine induced immunity, and the vaccine distribution. We fitted our model to COVID-19 cases among the vaccinated and unvaccinated, for <60 and 60+ age groups, and quantified the transmission rate, the vaccine efficacy over time and the impact of the third dose booster vaccine. The peak transmission rate of the Delta variant was found to be 2.14 times higher than that of the Alpha variant. The two-dose vaccine efficacy against infection dropped significantly from >90% to ~40% over 6 months. We further performed model simulations and quantified counterfactual scenarios examining what would happen if the booster had not been rolled out. We estimated that approximately 4.03 million infective cases (95%CI 3.19, 4.86) were prevented by vaccination overall, and 1.22 million infective cases (95%CI 0.89, 1.62) averted by the booster.

## Introduction

The COVID-19 pandemic caused tremendous impact globally, but would certainly have led to far more damage if effective vaccines against SARS-CoV-2 had not been rapidly developed and deployed. In addition, the emergence of new SARS-CoV-2 variants having higher transmissibility and potential for immune evasion [1] posed new challenges for mitigating the pandemic through vaccination. In this respect, Israel serves as an excellent case-study of vaccine effectiveness, as it has been a frontrunner in the vaccination campaign: the first-dose vaccination coverage, which began on 19 December 2020, exceeded 60% population coverage by March 22, 2021; and the second dose vaccination (fully vaccinated), began on January 9, 2021 and exceeded 60% population coverage by July 11, 2021. The vaccine was first prioritized for the elderly, while the government approved vaccination for all adolescents 11–18 since June 2021 [2, 3], and children from 5–11 years of age since November 14, 2021. The most commonly administered vaccine in Israel is the BNT162b2 vaccine, often referred to as the Pfizer vaccine. The vaccine has high estimated efficacy (95%) against infection and against severe outcomes from the wild-type strain [4]. However, despite the high vaccine coverage and purported efficacy, a large proportion of vaccine breakthrough severe cases unexpectedly appeared in Israel during August 2021 with the new invasion of the Delta variant. Fortunately, after July 31, the government had already begun providing a third “booster” dose of the vaccine, initially targeting the elderly [5], and by December 2021 approximately 80% of the eligible (4 million people in total) population had received the booster [6].

**Fig 1:**
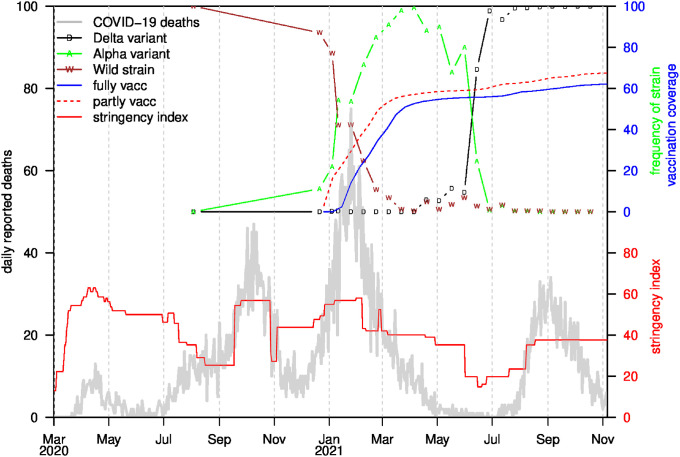
Daily reported COVID-19 deaths (grey) over pandemic period. Colored symbols show frequency of individual strains (as a percentage) of all strains sequenced (mostly biweekly) in Israel. Also plotted is the vaccination coverage (i.e. proportion of the population being vaccinated) with solid blue representing fully vaccinated and red dashes for partially vaccinated (one dose), bold grey curve for the daily reported COVID-19 deaths. The Alpha variant replaced the wild-type strain in December 2020-Febrary 2021, while the Delta replaced the Alpha variant in June-July 2021. The final wave in the graph is almost completely due to the Delta variant. The solid red curve represents the stringency index (see SI4).

The complex dynamics of the pandemic in Israel are illustrated in Fig 1, which shows the daily deaths as the pandemic unfolded in four waves from March 2020 to November 2021 (see Materials and methods for data sources). The evolution of the pandemic was controlled by “on-and-off” lockdown and mitigation measures (which may be quantified by a “stringency index”, solid red curve in Fig 1; see section 4 in S1 Text), strain replacement and the country’s increasing cumulative vaccination coverage. Three strains of SARS-COV-2 dominated at different times in Israel over this period: the wild-type strain before Jan 2021, the Alpha variant (B.1.1.7) between January 2021 and June 2021, and the Delta variant (B.1.617.2) after June 2021 [7]. The first two waves were caused by the wild-type strain, while the third wave coincided with the transition from the wild strain to the Alpha variant. As mentioned, the fourth wave included a large proportion of breakthrough infections (BTI), which occurred in parallel with the arrival of the Delta variant.

The emergence of a large number of BTI cases is due to the drop of vaccine effectiveness (VE) among a highly vaccinated population. The drop of VE is due to a combined effect of natural waning of immunity from previous vaccines or infection, and immune evasion associated with the invasion of a new variant, i.e., the Delta variant [6, 8, 9]. The focus of this paper is to quantify the drop of VE and thus estimate the effectiveness of the vaccination campaign.

The relative importance of natural waning vs. vaccine mismatching is not assessed here given the difficulties in disentangling the two effects from the observed VE measurements.

In addition to vaccination, the Israeli government issued various mitigation policies including a national lockdown from December 27, 2020 to February 5, 2021. But once the concurrent rapid vaccination campaign reduced the number of infections and deaths, the government removed most coronavirus restrictions. Nevertheless, the restrictions on gatherings were reinstated on July 29, 2021 when only people who were vaccinated or had recently tested negative were permitted to enter public places [10–12].

Several studies sought to examine the effectiveness of the BNT162b2 vaccine in Israel. Haas et al. [13] analysed national surveillance data in Israel between January 24 to April 3, 2021, and estimated an effectiveness against infection of approximately 95% at 7 days or more after the second dose. Goldberg et al. [9] compared the risk of infection in early July 2021, among the elderly (age 60+ years) who received the second dose of vaccine in March 2021 (four months after becoming fully vaccinated) to those who received the second vaccine dose in January 2021 (six months after becoming fully vaccinated). They found that the protection against infection decreased from 73% for those in the March group, to 57% for those in the January group, while the efficacy against severe symptoms deteriorated from 91% to 86% in the same setting. In the USA, Tartof et al. reported the VE of BNT162b2 to drop from 88% to 47% after 5 months [14].

As mentioned above, one of the key characteristics of the fourth wave in Israel was the emergence of the Delta variant. It was quickly understood that the Delta variant had enhanced transmissibility [15] and poorer prognoses [16] compared to the wild type strain. Accordingly, when the Delta variant dominated in July 2021, and few control measures were in place, the number of cases and hospitalizations increased significantly [5]. By September 13, 2021, among the 664 severe cases in Israel, 25% of the patients had received two doses of vaccine, 65% patients were completely unvaccinated, and only 8% had received the booster shot [17].

Vaccine effectiveness against the Delta strain has been vigorously investigated since the strain’s emergence. However, the effectiveness seems to be highly dependent on, and hard to disentangle from, the time elapsed since vaccination. For example, a study of the UK population, largely within four months of vaccination at the time, estimated effectiveness of two doses of the BNT162b2 vaccine against infection with the Delta variant at 88%. On the other hand, Israel’s Ministry of Health (MoH) reports estimated between 64% and 39% effectiveness [18], when observing the population approximately 5 and 7 months after vaccination [19]. This has been echoed in other studies in Israel, describing a combined effect of natural waning immunity and Delta strain invasion on vaccine effectiveness [8, 9]. In this work, by modelling waning of immunity we refer to modelling the combined effect of the natural waning immunity and vaccine escape.

In light of the studies above, the main drivers behind the COVID-19 wave and the observed high BTI ratio in Israel are still unclear. Hence, here we present a model which takes into account the age-classes of the vaccinated and unvaccinated population, the BTI ratio, the protection duration (and distribution) of vaccine induced immunity, time varying transmission rate, and the effectiveness of the second and third vaccination doses to examine this as closely as possible.

## Materials and methods

### Data

The following data sources, obtained from the Israel Ministry of Health (MOH) and from the World Health Organization (WHO), formed the basis of this modelling study.

1. Daily reported PCR-confirmed cases stratified by two age groups (<60 and 60+) and vaccination status between January 17, 2021 and October 27, 2021 [20].
2. Weekly reported PCR-confirmed cases stratified by age from March 21, 2020 until November 6, 2021 [21].
3. Daily number of COVID-19 hospitalizations and summary of gender, vaccination status, ventilator usage, and severity levels from March 11, 2020 until November 6, 2021 [22].
4. Daily vaccination with three doses stratified by age, from December 20, 2020 until November 6, 2021 [23].
5. Daily diagnosed severe cases stratified by age, including patients’ status of vaccination, from July 29, 2021 until November 6, 2021 [24].
6. Daily confirmed cases and deaths for all ages from March 1, 2020 until November 6, 2021 [25].
7. Proportions of the different variants of concern confirmed as they varied over time [26]. We downloaded aggregated variant proportion data from “The our world in data,” data that was originally obtained from GISAID [27–29].

The definitions relating to vaccination used here conform to those applied by Israel’s MOH [30]: individuals were considered fully vaccinated if they received 2 doses of vaccine and if 7 days had passed since the second dose; or if they had been infected with COVID-19 before they received a dose of the vaccine, and 7 days had passed since the first dose of the vaccine. Partially vaccinated individuals were those who received the first dose of vaccine but not the second dose and those who have received the second dose for less than 7 days. Unvaccinated individuals were those who had not received any vaccine dose.

### Model

The model formulation used here is an elaboration of the conventional SEIRV epidemic population model where the variables *S*, *E*, *I*, *R* and *V*_(*k*)_ denote the number of individuals in the Susceptible, Exposed, Infectious, Recovered and Vaccinated groups [31, 32]. Susceptible individuals flow through the compartments in linear order: *S→E→I→R*. A proportion ${\tilde{v}}_{i}(t)$ of susceptible individuals are shunted to the vaccination compartments. As explained in more detail shortly, in order to model the combined effects of natural waning of immunity and vaccine escape, the vaccinated class *V*_(*k*)_ is broken down into a series of *n*_*V*_ = 5 stages (*k* = 1,2,3,4,5; see [33]). The model includes age-classes with *i* = 0,1 indicating <60 years and 60+ years, respectively. The population of Israel was approximately 9.28 million people in 2021, and the 60+ age group accounted for 16.4% of this number. The two age classes were chosen on the basis of data availability (see datasets above) and due to the interest here in the elderly component of the population.

A special feature of the model is the incorporation of BTIs, which arise in those vaccinated individuals in the *V*_(*k*)_ (vaccinated) group where they at first gain medium-term temporary immunity. When this protection wanes, they join the *S*_*V*_ class of post-vaccination susceptible and become susceptible once more, and have the potential to generate BTI’s. We assume those individuals in *S*_*V*_ have partial protection (i.e., reduced susceptibility) to infection, as controlled by the parameter *ε*. Upon infection, they join the breakthrough exposed class, *E*_*B*_. For simplicity, we did not track BTIs after their infection, and just allowed individuals in *E*_*B*_ to join the *I* class after their latent period. Realistically however, these BTIs have reduced transmissibility when infected. This effect was modelled by introducing one more parameter, *ω*, which reduces transmissibility in BTIs (see section 2 in S1 Text). The effect of the booster (third dose) vaccine is modelled by shunting a proportion $\alpha \tilde{b}(t)$ of individuals in *V*_(*k*)_ and *S*_*V*_ into the *R* class. The term $\tilde{b}(t)$ is the daily proportion of fully vaccinated individuals receiving the third dose, whereas *α* represents the vaccine efficacy (VE) of the third dose. We assume *α* ≈ 1 for a short period after the booster [5, 8] (and also test this assumption (see SI)).

The full model is given by the following ordinary differential equation system:

$$S_{i}^{\prime }=-\beta (t)(I_{i}+I_{1-i})S_{i}-{\tilde{v}}_{i}S_{i},$$

$$V_{i\left(1\right)}^{\prime }=\eta_{2}{\tilde{v}}_{i}S_{i}-\;n_{V}\kappa V_{i\left(1\right)}-\alpha \tilde{b}V_{i\left(1\right)},$$

$$\begin{array}{l} V_{i\left(k\right)}^{\prime }=n_{V}\kappa V_{i\left(k-1\right)}-n_{V}\kappa V_{i\left(k\right)}-\alpha \tilde{b}V_{i\left(k\right)},\\ \ldots \end{array}$$

$$S_{V,i}^{\prime }=\eta_{3}{\tilde{v}}_{i}S_{i}+n_{V}\kappa V_{i\left(n_{V}\right)}-\varepsilon \beta \left(t\right)\left(I_{i}+I_{1-i}\right)S_{V,i}-\alpha \tilde{b}S_{V,i},$$

$$E_{i}^{\prime }=\beta \left(t\right)\left(I_{i}+I_{1-i}\right)S_{i}-\sigma E_{i},$$

$$E_{B,i}^{\prime }=\varepsilon \beta \left(t\right)\left(I_{i}+I_{1-i}\right)S_{V,i}-\sigma E_{B,i},$$

$$I_{i}^{\prime }=\sigma \left(E_{i}+E_{B,i}\right)-\gamma I_{i},$$

$$R_{i}^{\prime }=\gamma I_{i}+\eta_{1}{\tilde{v}}_{i}S_{i}+\alpha \tilde{b}V_{i\left(1\right)}+\cdots +\alpha \tilde{b}V_{i\left(5\right)}+\alpha \tilde{b}S_{V,i}.$$

**Fig 2:**
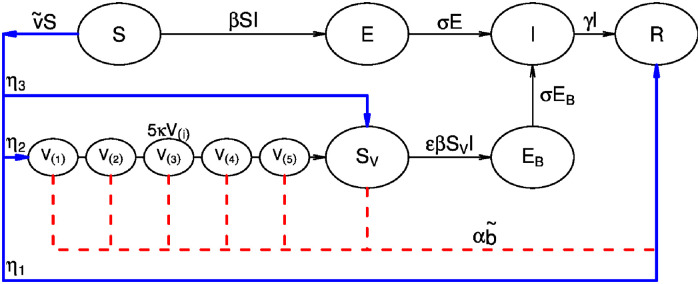
A flowchart of Susceptible-Exposed-Infective-Recovered-Vaccinated (SEIRV) model for one age group. Susceptible individuals flow through the compartments in linear order: *S* → *E* → *I* → *R*. Vaccinated susceptible individuals are of three immunity types: a proportion (*η*_1_) move directly to the Recovered class and gain long-term protection; a proportion (*η*_2_) gain medium-term temporary protection and move through a series of five *V*_(*k*)_ classes. After a period in which immunity wanes, they then move on to the *S*_*V*_ class. Individuals in *S*_*V*_ are susceptible to breakthrough infections and move to the *E*_*B*_ and I class; a proportion (*η*_3_) of those vaccinated have little protection and move to *S*_*V*_ class directly. A proportion of individuals in *V*_(*k*)_ and *S*_*V*_ receive the booster dose and move to the *R* class. Bold blue lines indicate full vaccination (second dose) and dashed red lines indicate booster vaccination.

The parameters were set to reflect the most current knowledge of COVID-19 dynamics. In particular, exposed individuals move to the infectious class after a mean latent time of 1/*σ* = 2 days. Infected individuals remain infectious for a mean time of 1/*γ* = 3 days [34–36]. This gives a short mean generation time of 5 days, which is the sum of the mean latent period and the mean infectious period. The model takes into consideration imperfect reporting, by assuming that only a proportion *ρ*_*i*_ of infected individuals are reported in each age-class *i*. The model infers *ρ*_*i*_ with the assumption that *ρ*_*i*_ ≥ 0.4 to reflect the high medical accessibility rate in Israel. A schematic flowchart of the model is given in Fig 2.

### Time varying transmission rate

Over the study period, the country’s mitigation measures, public risk perception, seasonality, and behaviors changed (as reflected in the stringency index, see SI4), and thereby continuously modified the contact rates, and thus ultimately the transmission rate. Additionally, the invasion of variants having increased transmissibility are able to modify the transmission rate in our model. Hence, these changes need to be reflected through a time varying transmission rate *β*(*t*), (following our previous work [36]). In practice, *β*(*t*) is obtained in the process of fitting the model to the weekly reported cases (in four categories: vaccinated and unvaccinated, in <60 and 60+) using an exponential cubic spline [37–41] with *n*_*β*_ = 13 nodes over the study period of 85 weeks, namely 6.5 weeks per node. Since there were more fluctuations in 2020 than in 2021, we allocate 9 nodes evenly distributed before December 2020, and four nodes evenly distributed after December 2020. Note that the time varying basic reproductive number (immunity-unadjusted) is given by $R_{0}\left(t\right)=\beta \left(t\right)/\gamma$ in the absence of vaccination, and is a measure of the transmission rate. The immunity-adjusted reproductive number (i.e., the effective reproductive number, termed $R_{e}\left(t\right)$ or $R_{t}\left(t\right)$ sometimes) is $R_{e}\left(t\right)=R_{0}\left(t\right)(S\left(t\right)+\varepsilon S_{V}\left(t\right))$.

### Modelling vaccination

#### Immunity waning of the second dose

In the classic SEIR model, the duration time of individuals remaining in each compartment is exponentially distributed. However, the duration which vaccinated individuals spend in the *V* compartment is unlikely to be exponentially distributed, since waning does not appear to decline exponentially. Therefore vaccinated individuals in our model were passed through a chain of five serial compartments so that the duration in the *V* classes follows the very flexible gamma distribution [42] whose parameters (and thus shape) can be fitted.

In more detail, vaccinated susceptible individuals were divided into three groups:

1. a proportion (*η*_1_) gain long-term protection and move directly to the recovered R class;
2. a proportion (*η*_2_) gain medium-term temporary protection by passing through a series of five *V*(_*k*_) classes, during which immunity wanes slowly (set by 1/*κ* year), and then finally move on to the *S*_*V*_ class. Individuals in *S*_*V*_ are susceptible to breakthrough infections and move to the *I* class through an exposure stage (*E*_*B*_);
3. and the remaining proportion (*η*_3_) gain little protection from vaccination and move to *S*_*V*_ directly.

By definition, the proportions *η*_1_ + *η*_2_ + *η*_3_ = 1. These parameters are difficult to estimate separately only from observational data, and so their values have to be approximated to some extent to match observed characteristics of the vaccine. We set *η*_1_ = 0.1 to emulate that a small fraction of individuals do not lose immunity (at least in the period modeled in this work), as some level of protection appears to remain over long time periods in waning immunity studies [9]. We set *η*_3_ = 0.1 as 1 minus the approximate short term VE (~90%) estimated from the original vaccine randomized control trials [4]. (In the other words, the immediate risk of infection shortly after second dose vaccination is 10%.). Hence, we set *η*_2_ = 0.8.

As mentioned, for a simple model, if there were only a single vaccination compartment, vaccine-induced immunity is usually set to wane exponentially. However, for the full model presented here, by adding a chain of vaccination compartments, the waning is modified to follow a more realistic Gamma distribution, which reflects the possibility that the vaccine might be highly effective at least over the first months. For five stages, the waning has a Gamma distribution with shape parameter *n*_*V*_ = 5. The rate parameter of the Gamma distribution can be denoted as *n*_*V*_*κ*. The mean of the Gamma distribution is nVnVκ=κ-1, which is independent of *n*_*V*_. The VE can be expressed as:

$$VE=\eta_{1}+\eta_{2}\left[1-F\left(\kappa ,\tau \right)\right]+[\eta_{2}F\left(\kappa ,\tau \right)+\eta_{3}](1-\varepsilon ),$$

where *F* is the cumulative distribution function of individual protection duration (which is Gamma distributed) for those in the *V* classes [43].

The parameter *τ* is the time elapsed since the second dose vaccination.

The parameter *ε* reflects the susceptibility of vaccinated individuals to BTI. When *ε* = 1, vaccinated individuals are as susceptible to infection as unvaccinated. In contrast, when *ε* = 0, vaccinated individuals have permanent immunity and can never be infected. Initially *τ* = 0, and the efficacy is *η*_1_ + *η*_2_ + *η*_3_ (1 − *ε*), which over time decreases to a level of *η*_1_ + [*η*_2_ + *η*_3_](1 − *ε*) for large *τ*. Under our parameter values, these two levels are 92% and 28% for large *τ* with *ε* = 0.8. In our baseline model, with parameters *ε* = 0.8, *κ*^−1^ = 3.75 months (as determined from fitting the model—see Results) and *n*_*V*_ = 5. The VE also begins at 92% and a calculation shows that in our model it declines to 39% in the six remaining months of the study period. This corresponds to the VE of 39% reported by Israel’s MOH in July 2021 some five months after the second vaccine program was initiated and vigorously pushed out.

When vaccination is modelled with a single stage and thus exponentially distributed (i.e. *F* = 1 − *e*^*−κτ*^), a simpler more intuitive form is obtained

$$VE=\eta_{1}+\eta_{2}e^{-\kappa \tau }+[\eta_{2}(1-e^{-\kappa \tau })+\eta_{3}](1-\varepsilon ).$$

### Vaccine efficacy of the third dose (*α*)

The model takes into careful account waning of the first and second vaccine doses. Based on the study’s timeframe, it was not necessary to include third dose waning. The VE of the third dose was set as *α*, which is the proportion of the boosted population who gained long term protection (i.e., protection lasting beyond the end of the study period). The remaining 1- *α* proportion of the boosted population gained zero protection.

### Breakthrough infections

We use the breakthrough infection (BTI) ratio as a key index in this work. The BTI ratio is defined as the ratio of the number of active BTIs among the active infectious (including both BTIs and non-BTIs), at a given point in time for an age group. It is defined for the *i*’th age-group as:

$$BTI\_Ratio_{i}\left(t\right)=E_{B,i}\left(t\right)/\left(E_{U,i}\left(t\right)+E_{B,i}\left(t\right)\right),$$

where *i* = 0,1 represents populations <60 and 60+ years of age, respectively; *E*_*B*,*i*_ is the number of exposed cases due to BTI, i.e. among the vaccinated; and *E*_*U*,*i*_ is the number of exposed cases among the unvaccinated. BTIs generally lead to milder symptoms than infections amongst the unvaccinated in the same age-group [44, 45]. Thus, the ratio of BTI severe cases among all severe cases, denoted as *BTI*_*Ratio*_*i*__severe, should be smaller than the BTI ratio amongst all infected individuals. That is, *BTI*_*Ratio*_*i*__severe < *BTI*_*Ratio*_*i*_.

### Technical considerations

Several additional technical modelling considerations have to be dealt with and these are discussed in SI2. For example, the model also assumes that there is a 7-day delay between the date of the second vaccination dose delivered (i.e, 28 days after the first dose, and assuming a 21-day gap between the first dose and the second dose) and the onset date of the protective effect [46]. This is implemented by incorporating a time delay whereby the vaccination rate ${\tilde{v}}_{i}\left(t\right)$ calculated from the data (see SI2) is updated and replaced by ${\tilde{v}}_{i}\left(t-7\right)$. The reduced transmissibility of BTIs, as compared to non-BTIs, is discussed in SI2. The booster dose vaccination rate ${\tilde{b}}_{i}\left(t\right)$ is calculated from the data, as described in SI2.

Reinfection is now understood to play a weak role in the dynamics of the outbreaks modelled [47]. For the sake of model tractability it is reasonable to assume its role is negligible in the model.

### Model fitting

The plug-and-play likelihood-based inference framework which was implemented in the R package POMP [48, 49] was used. The model was run using the Euler-multinomial integration method, which takes into account demographic noise. A negative-binomial measurement model was used to link model simulated weekly cases and observed weekly cases to take into account measurement noise. These methods have been thoroughly described in [50, 51]. Overall, we estimate 17 parameters in our baseline model, including 13 parameters for the transmission rate, and four extra parameters (*ρ*_1,2_, *κ*, and *τ*), besides initial conditions (see Table B in S1 Text).

## Results and discussion

### Epidemic dynamics captured by the data

**Fig 3:**
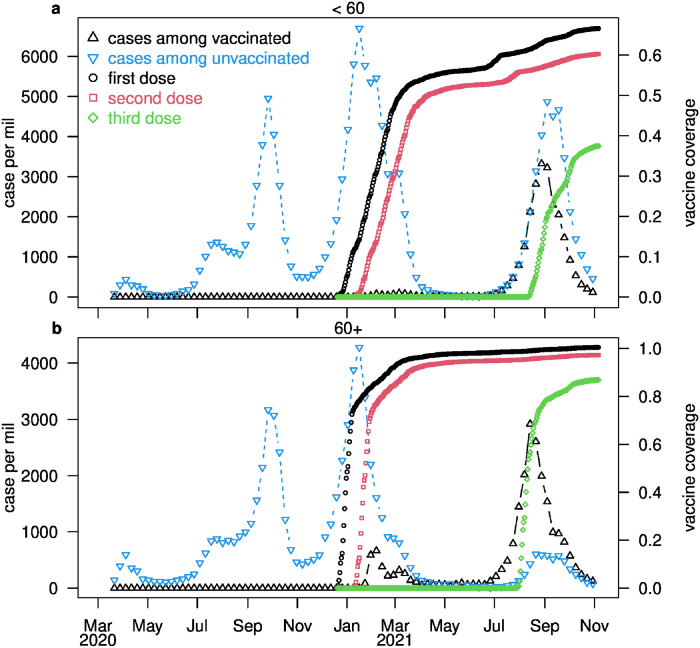
Infection and vaccination dynamics in Israel. Weekly reported COVID-19 cases are presented for the <60 age group in (a) and for the 60+ age group (b) (population standardized, per 1 million individuals in an age group) in unvaccinated (blue, down-facing triangles) and fully vaccinated (black, up-facing triangles) individuals. The cumulative vaccine coverage for the first, second and third dose are represented by black circles, red squares, and green diamonds. respectively.

Fig 3 presents the reported weekly confirmed COVID-19 cases in Israel divided into vaccinated and unvaccinated, for age groups <60 years (panel a) and 60+ years (panel b). The case numbers are standardized and given per 1 million individuals in an age group. Superimposed on the figure is the vaccine coverage for the first, second and third dose, respectively. A more detailed breakdown into age-classes is given in Fig A in S1 Text. The vaccination program was initiated close to New Year 2021. The number of reported cases increased significantly from December 2020 to January 2021, reaching the largest number of daily infections ever experienced in Israel, when the epidemic rapidly declined from January until June. The country’s mitigation and lockdown policies could have played a large role in the epidemic crash. The crash was also simultaneous with the first distribution of the vaccine, which began in Israel in December 2020, initially targeting the elderly population [52].

Some 68% of the 60+ age group were vaccinated in January 2021. This helps to explain why the epidemic curve for the 60+ population appeared to respond more quickly, as its trajectory decreased over January. Thus, the vaccination program is likely to have been at least partially responsible for the demise of the outbreak.

By July 2021, more than 60% of the Israeli population were fully vaccinated. Moreover, the country had already experienced three major waves of the pandemic and thus reached a large total attack rate by this point in time. Taken together, these two facts imply that the number of susceptible people remaining in Israel should have become quite limited, explaining why the disease appeared to have become almost eradicated. Despite the appearance of eradication, in late June a surprising fourth wave struck Israel. The data suggest that the three key factors responsible for the fourth wave are i) the dominance of the highly transmissible Delta variant, which completely replaced the Alpha variant in Israel; ii) the large numbers of BTIs that began to appear, either due to the waning of immunity or Delta variant vaccine escape (note that we used waning of immunity to refer the effects of both natural waning and vaccine escape.); and iii) relaxation of control measures during the summer and pandemic fatigue of the public. The relaxation occurred in many countries, due to the wide implementation of vaccination, and downtrend of the global pandemic; it was hoped the pandemic was finally over. All these together triggered the new Delta wave.

Lastly, notice that the per million booster vaccine coverage over the Delta wave was less than half in the <60 age group than that of the 60+ age group (see Fig 3). The number of BTIs per million, however, was similar for the two age groups. However, the Delta wave was characterised by a very large number of cases among the unvaccinated in the <60 age groups, reaching a peak of >4,800 cases per million per week in August 2021, as compared with a peak of ~600 cases per million per week in the unvaccinated >60 age class. Thus, the fourth wave in Israel was to an extent a result of low vaccination coverage amongst the <60 year age class.

### Fitting the outbreak dynamics

**Fig 4:**
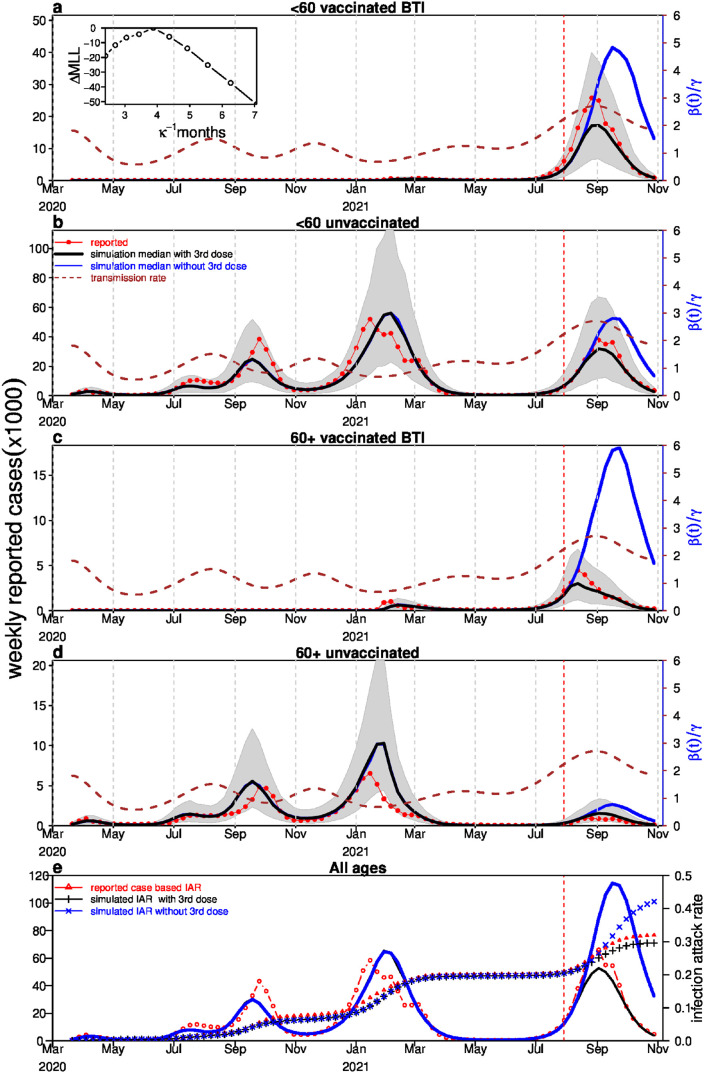
Model fitting results. The simulated cases (black bold) versus observed cases (red circles) for the <60 age vaccinated group in (a); for the <60 unvaccinated group in (b); for the 60+ vaccinated group in (c); for the 60+ unvaccinated group in (d) and for all groups in (e). The cases in (a,c) are BTIs. The brown dashed curve shows the time-varying basic reproductive number (i.e., a scaled transmission rate). The inset in panel (a) shows the profile maximum log-likelihood as a function of *κ*, and used to determine the best estimate of *κ*. The blue curves show the counterfactual scenario where the effect of the third dose is shut off (by letting *α* = 0). The vertical dotted red line indicates the initiation of the booster dose. The infection attack rates (IAR) based on reported cases, simulated cases with booster and simulated cases without booter are denoted as red curve with triangles, black curve with plus and blue curve with cross.

Here we use the baseline model to investigate the above dynamics by fitting the observed epidemic data over all four waves of the pandemic, during the full study period, from March 2020. Specifically, the model is used to fit the four time series seen in Fig 4: infected cases amongst those who were vaccinated (i.e., BTI’s) and infected cases amongst those who were unvaccinated, for each of the two age groups. The vaccination rate of the second doses, ${\tilde{v}}_{i}(t)$, and the booster dose, ${\tilde{b}}_{i}(t)$, for the two age groups, were determined from the data in Fig 3a & 3b.

**Fig 5:**
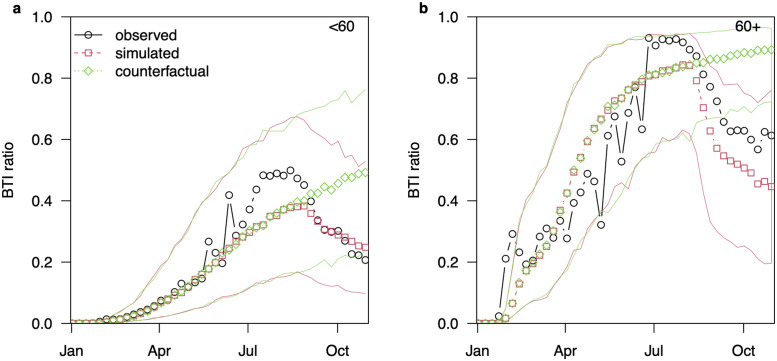
The observed and simulated BTI ratio in 2021. The observed (black circles) versus the simulated BTI ratios under the baseline, *α* = 1 (red squares) and counterfactual, *α* = 0 (green diamonds) scenarios for <60 age group in panel (a) and for 60+ age group in panel (b). These results correspond to Fig 4, showing the BTI ratio instead of case numbers. Red and blue curves indicate the 95% CI ranges for each scenario.

The model fits are displayed in Figs 4 and 5. The simulated case numbers provide an excellent fit to the reported case numbers in all four groups: <60 vaccinated (a), <60 unvaccinated (b), 60+ vaccinated (c), and 60+ unvaccinated (d), and in all groups overall (e), over all four waves of the epidemic. The simulated infection attack rates (cumulative proportion of population who were infected) with the booster and without booster are given in (e). The reconstructed time-varying basic reproductive number $R_{0}\left(t\right)=\beta \left(t\right)/\gamma$ is plotted as a brown dashed curve. $R_{0}\left(t\right)$ peaked in July 2020 and December 2020, and then decreased. Presumably, the peak in December can be attributed to kindergartens and schools being gradually reopened since the end of October, while the decrease is plausibly due to the restrictions of social distance and the nation-wide lockdown implemented by the Israeli government [53–55]. In June 1, 2021, Israel revoked almost all the restrictions, apart from mask wearing indoors, which helps explain why the transmission rate in Fig 4 kept increasing [56]. We note that our estimated $R_{0}\left(t\right)$ is consistent with empirical estimates by the Israeli MoH until vaccines were deployed in Israel, when the estimates diverge and $R_{t}$ becomes substantially lower (since $R_{0}\left(t\right)$ does not account for vaccination, i.e, immunity-unadjusted). The correspondence between the two estimates is shown in Fig E in S1 Text.

Finally, the time varying $R_{0}\left(t\right)$ is plotted in Fig 4 (dashed brown curve), and gives an indication of the transmissibility of the Delta variant between August and October 2021. In particular it can be seen that $R_{0}\left(t\right)$ peaks by 2.14-fold relative to its peak value during Alpha dominate period. In Fig 5, we show the BTI ratio based on the case numbers in Fig 4.

Through the model fitting, the temporary duration of protective immunity from vaccination, or “zero susceptibility” stage, of those for whom the second dose vaccine is effective but wanes (the fraction *η*_2_), was estimated as *κ*^−1^ = 3.75 months (95% confidence interval 3.3, 4.14) (Fig 4a, inset). The vaccine efficacy begins at 92% in January 2021, and the model fit determined it declines to 35% over six months. This is comparable to estimates from the literature where immunity is found to wane substantially between two and six months [9] after second dose vaccination, and reports of reaching 39% have been published [18].

The reporting ratios of cases *ρ*_*i*_ were found to be approximately 44.57% and 50.22% for the two age groups, respectively, which are epidemiologically reasonable (see [6]). Namely, for any reported case, there was an additional unreported case. Our estimated infection attack rate was 3.9% by September 11, 2020, which is in line with the seroprevalence of 4.6% estimated by Reicher et al. [57].

Of particular interest is that the Delta variant invaded Israel some 4–5 months after the second dose of the vaccine was distributed to the population on a large scale. That is, just when the vaccine immunity was beginning to wane. At that point, the Delta variant outbreak increased the BTI ratio which peaked at 85% for the 60+ age group at the end of August 2021, and then dropped sharply. This can be contrasted with the BTI levels observed if the third dose (booster) is removed by letting *α* = 0 (see Fig 5). Note that in Fig 4, the baseline model with *α* = 1 is fitted to the observed data. The counterfactual scenario presents a simulation of the baseline model where the only parameter differing from the baseline model is *α* = 0.

Together, the two effects of the invasion of the Delta variant and implementation of the third dose, account for the observed BTI pattern. However, the exact contribution of immunity waning and the increased transmissibility, or immune escape, of the Delta variant is difficult to disentangle given the current data. We compared our estimated VE with the results of Goldberg et al. [9] and Chemaitelly et al. [58] in section 5 in S1 Text.

### Breakthrough infections

**Fig 6:**
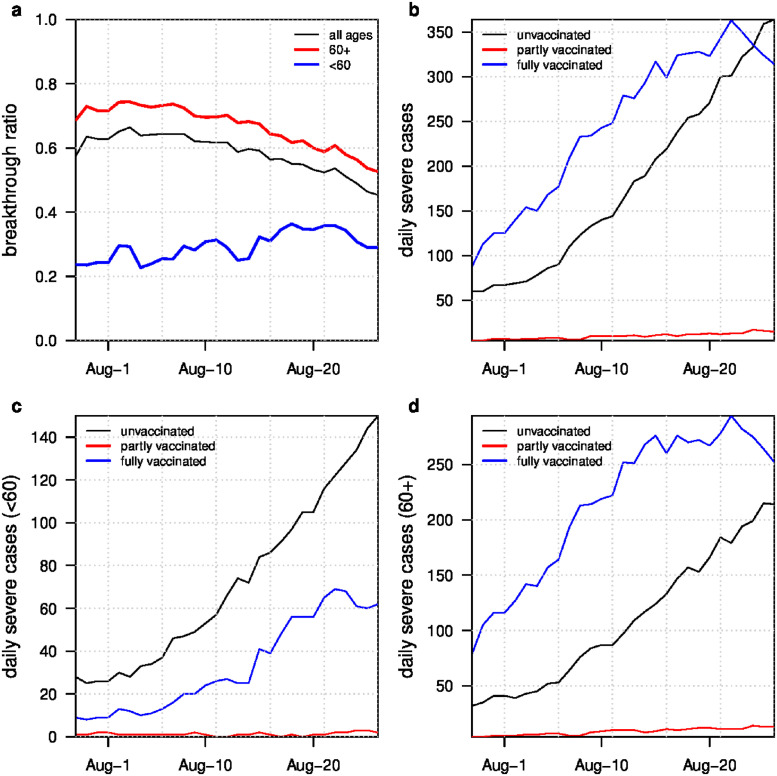
(a) Breakthrough infection ratio among severe cases (*BTI*_*Ratio*_*i*__severe) over time for all ages, age 60+ and age <60. (b) Daily reported severe cases for three types (unvaccinated, partly vaccinated, fully vaccinated) for all ages over time. (c) three types of daily severe cases for <60 years old. (d) three types of daily severe cases for 60+ year olds.

Fig 6 shows the BTI dynamics during August 2021. In Fig 6a, we can see that *BTI*_*Ratio*_*i*__severe in the 60+ age group is greater than 0.6 for most of August 2021. As mentioned above, since BTI cases should have relatively mild symptoms, the BTI ratio amongst all infections (both severe cases and mild cases) should be no less than 0.6 among the 60+ age-group.

We observe that the BTI among all cases in the 60+ group reached 85% in Fig 5b. Furthermore, there were approximately 60% BTIs among severe cases between July 29, 2021 and August 20, 2021 (Fig 6). In other words, if *n* individuals were infected, 0.85*n* would have been vaccinated. If a proportion *q* of the infected developed a severe disease, 0.6*q* of them would have been vaccinated. Hence the relative risk of severe outcome among BTI compared with unvaccinated is $\;\frac{0.6q}{0.85n}/\frac{0.4q}{0.15n}\cdot 100=\;26.5\%$. Therefore, given a breakthrough infection, the VE was 73.5% against severe outcome over the aforementioned period.

### Counterfactual model with booster “switched off”

We generated simulations from a counterfactual model, in which the third booster was removed or “switched to zero”. This was achieved by running the baseline model with the same parameters (including the same $R_{0}\left(t\right)$) apart from setting the vaccine efficacy of the third dose, *α*, to zero. The model thus simulated the number of infections expected in the absence of the booster. The counterfactual time series in this scenario (Fig 4, blue curves) unsurprisingly reveals a larger epidemic peak than that observed (Fig 4, red curves), especially for the 60+ population.

The cumulative number of infections under the counterfactual scenario can be compared with that of the baseline model to find the number of infections prevented by the third dose vaccination. Under this scenario, we estimated that the third dose vaccination prevented approximately 1.22 million infections (95%CI 0.87, 1.57). A logarithmic scaling version of Fig 4 is presented in Fig B in S1 Text and provides a zoom in on the dynamics.

In our baseline model, we assumed a reduced infectivity of BTI, *ω* = 0.8. If we relax this assumption and set *ω* = 1, the infections averted by the booster would be 1.48 million (95%CI, 1.11, 1.87).

We emphasize that this counterfactual scenario should be interpreted as a reasonable approximation only, as it assumes that all other factors would have remained constant. It is likely, however, that a sharp increase in infections would have led to more governmental restrictions, in turn reducing infection rates, thus these numbers might be over-estimates.

In the above, we consider the scenario of switching off the booster. Similarly, we can switch off all three vaccine doses. In this way, we found that the vaccination program (including all three doses) prevented 4.03 million infections (95%CI 3.19, 4.86), i.e., about 43.8% of the Israeli population. The corresponding infection attack rates (IAR), or proportions of population being infected by November 2021 under the actual and counterfactual scenarios are 31.7% and 75.5%, respectively (since 75.5%-31.7% = 43.8%). This level of infection is epidemiologically plausible. We note that only 10% of the Israeli population were infected by the end of 2020, given $R_{0}=2\;\sim 3$ for the wild strain and Alpha variant. This spreading speed is far less than a constant $R_{0}=2\;\sim 3$ would predict partly due to the presence of various NPIs. Without vaccination, our model suggests that an additional 65.5% of the Israeli population could have been infected by November 2021, thus reaching an infection attack rate of ~75.5% by the end of 2021. The vaccination campaign thus prevented 66.8% of infections (100*(1-(31.7–10)/(75.5–10))%).

The infection attack rate (IAR) by July 2021 was 21.2%, and it increased by 10.5% between July 2021 and November 2021. Gavish et al. [6] estimated a 5-fold increase if the booster vaccine was not rolled out, which means the IAR would have reached about 73.7% (= 10.5*5+21.2). While for our model, the IAR would have been 44.7% (= 10.5*2.24+21.2; see Fig 4e).

### Reinfection

In our model, we ignored reinfection. Before the emergence of the Omicron variant in December 2021, reinfection was relatively low. The cumulative cases until Jan 2021 constituted about 5% of Israel’s population [59], and reinfections were relatively rare—estimated < 1% in Israel [47, 60]. Moreover, the viral shedding of reinfected cases is substantially lower than primary cases, and thus the overall contribution of reinfection in forming the multiple waves was negligible [61, 62].

## Conclusion

In summary, we proposed a model framework analyzing COVID-19 dynamics in Israel that sheds light on important issues of BTI and waning immunity (including both natural waning and vaccine escape caused waning). Through mathematical modeling, we showed that the fourth wave of COVID-19 in Israel can mostly be explained by low vaccination coverage among <60 years individuals, the large proportion of BTI among vaccinated age 60+ years individuals (reaching levels of 85%), and a compromised vaccine efficacy due to a combined effect of immunity decay and the invasion of the Delta variant. Through a model reconstruction of the transmission rate, it was found that the peak transmission rate of the Delta variant was 2.14 times larger than the peak transmission rate of previous Alpha variant. The increased transmissibility may have been due to a combination of human behaviours and attributes of the Delta strain. The vaccine efficacy began at 92% in January 2021 but the model estimated it must have dropped to 39% 5–6 months later. We also presented a BTI analysis, showing that the BTI ratio indeed peaked during the Delta strain invasion, and before the third booster.

Furthermore, we simulated a counterfactual scenario where the third vaccine dose, or booster, was precluded. Under such a scenario, 2.2 million new infections would have occurred had there been no further governmental interventions in the period from July 2021 to November 2021. Given the observed number of infections was approximately 0.98 million (55.4% lower than the counterfactual), this implies that approximately 1.22 million infections were averted by the booster vaccine. Hence, the significance of the booster in mitigating the Israeli COVID-19 epidemic cannot be overstated.

This result should be contrasted with the very recent modelling results of Gavish et al. [6] who found that the observed infections were ~80% lower than the no-booster counterfactual over the period July 2021 to November 2021. Thus, their counterfactual model predicted that approximately 4.36 million inhabitants would have been infected had the booster vaccine not been rolled out, and 3.38 million infections were prevented by the booster vaccine. An early version of this paper was posted online on Jan 10, 2022, while an early version of Gavish et al. [6] was posted online on Jan 18, 2022.

Thus, our study found that although the booster was very effective, it averted about one third of the infections claimed in Gavish et al. We believe this is because of the different methods used for estimating the transmission rate over the epidemic. The method applied here fits a time varying rate that should reflect the transmission of the virus, including inferred impacts of mitigations. The transmission was found to vary consistently over four COVID-19 waves. In contrast, the Gavish et al. study [6] fits only a single (Delta) wave with transmission largely based on an assumed mobility index, that was in the end almost constant over the Delta wave, to some extent reliant on initial conditions and substantially larger than that found here.

Our analyses were applied to Israel—which has a highly vaccinated population, and an effective health care system with an efficient and publicly available data collection system. It therefore served as an excellent case study, especially given its early drive to hastily redistribute the booster dose. Thus, the lessons learned from the dynamics in Israel may serve to increase preparedness in other locations observing invasions of variants of concern. In the future, our proposed framework can also be applied to new data on emerging variants, to compare and examine different scenarios as this epidemic or other outbreaks unfold.

## Supporting information

S1 Text (DOCX)
