# Supplementary material for: Modelling COVID-19 vaccine breakthrough infections in highly vaccinated Israel—The effects of waning immunity and third vaccination dose
Source: PLOS Glob Public Health. 2022 Nov 9;2(11):e0001211. doi: 10.1371/journal.pgph.0001211 (PMC10021336; doi:10.1371/journal.pgph.0001211)
Supplement: S1 Text — (DOCX) [file pgph.0001211.s001.docx]

Modelling COVID-19 vaccine breakthrough infections in highly vaccinated Israel – the effects of waning immunity and third vaccination dose

Anyin Feng, Uri Obolski, Lewi Stone, Daihai He

**S1 Text**

**Section 1: Vaccine coverage of the three doses among age groups**

Figure A shows the vaccine coverage in Israel from December 20, 2020 to November 6, 2021. The vaccine priority was for the elderly first in December 2020, while at the last stages the government approved all adolescents 11-18 to vaccinate beginning in June 2021 (1, 2), and the children from 5-11 years of age since November 14 2021. The ages of those receiving the booster third dose of vaccination (Fig A panel c) were mainly adults between 40 and 69 years old. Compared with this age group, older people and younger people were poorly vaccinated.

**Figure A.** Vaccination coverage stratified by age group in three scenarios. (a) LH panel: First dose (partly vaccinated) (b) Middle panel: Second dose (fully vaccinated) (c) RH panel: Third dose. In the coverage in the age group <20 year is much lower than other age groups.

The gap between the first dose and the second dose is 21 days for most receivers. And it takes 1 to 2 weeks for the first dose to elicit protection. Thus the partial protection provided by the first dose during the gap between the first dose and the second dose is ignored in our model for simplicity.

**Section 2: Technical issues**

***a) Reduced infectivity of Breakthrough Infection cases.*** The BNT162b2 vaccine reduces the infectivity of vaccinated individuals (3). The reduced infectivity ($\omega<1)$ of BTI cases can be modeled as:

$$\beta\left( I_{U}+{\omega I}_{V} \right)=\beta\left( \frac{E}{E+E_{V}}I+\omega\frac{E_{V}}{E+E_{V}}I \right)=\beta I\frac{E+\omega E_{V}}{E+E_{V}}$$

Where $I_{U}\mathrm{and}I_{V}$represent infections among unvaccinated and vaccinated, respectively. Thus in model equations (1), we replace $\beta$ with $\beta\frac{E+\omega E_{V}}{E+E_{V}}$. Based on (4), we assume $\omega=0.8.$

***b) Vaccinating by targeting the unvaccinated:***  The proportion of the whole population that becomes fully vaccinated each day is denoted $v_{i}\left( t \right)$, for age-group $i$. This rate is easily calculated given the cumulative proportion of the population that have been vaccinated daily. In reality, each age group of the population is divided into two sub groups, those vaccinated and those not yet vaccinated, and only the latter group are eligible for vaccination, where susceptible belong to the latter group. To take this into account, the rate at which susceptible were vaccinated, $\tilde{v}_{i}$, for each age group is given by:

$\tilde{v}_{i}\left( t \right)=v_{i}(t)/(1-\int_{0}^{t-1} v_{i}(s)ds)$.

where the denominator is the proportion of the population that is unvaccinated. This conversion is important. One cannot use $v_{i}(t)$ directly, since the unit of $v_{i}\left( t \right)$ is per capita of the whole population per day, rather than per unvaccinated per day, while the vaccination targets the unvaccinated group. The model also assumes that there is a 7-day delay between the date of the second vaccination dose delivered and the onset date of the protective effect. Thus the peak of VE occurs at four weeks after the first dose (assuming a 21-day delay between the first dose and the second dose). This is implemented by incorporating a time delay whereby the vaccination rate $\tilde{v}_{i}\left( t \right)$ calculated from the data is updated and replaced by $\tilde{v}_{i}\left( t-7 \right)$. Similarly, we denote $b_{i}(t)$ the booster dose deliver rate per capita. Hence the rate among two dose fully vaccinated groups is

$\tilde{b}_{i}\left( t \right)=b_{i}(t)/(\int_{0}^{t-29} v_{i}\left( s \right)ds-\int_{0}^{t-1} b_{i}\left( s \right)ds)$.

That is, the third dose will be delivered to those who have received the second dose at least four weeks ago. This conversion yields the rate of the third dose among those individuals. However, this $\tilde{b}_{i}\left( t \right)$ will require the V class to be divided into two subclasses: those who received the second dose in the most recent four weeks, and those who received second dose outside the recent four weeks. For the sake of simplicity, we avoid further dividing of the V class, and assume everyone in the V class is eligible for the third dose. This increases the pool of third dose receivers slightly. To compensate, we reduce $\tilde{b}_{i}\left( t \right)$ by dropping the rule of four weeks,

$\tilde{b}_{i}\left( t \right)=b_{i}(t)/(\int_{0}^{t-1} v_{i}\left( s \right)ds-\int_{0}^{t-1} b_{i}\left( s \right)ds)$.

Unlike the first/second dose, the VE of the booster peaks almost immediately after the delivery of the booster. Thus no delay is needed when incorporating $\tilde{b}_{i}\left( t \right)$ into the model (5).

**Section 3: Sensitivity analysis and model selection**

Figure B shows the results of Fig 4 plotted with a logarithmic transformation.

**Figure B**. Replicate of Fig 4 of the main text on a logarithmic scale.

In Table A, we summarize the results of our sensitivity analyses. We found that

1. Case #1 to 3: Reducing $n_{V}$ (the number of stages in V) from 5 to 3 to 1, decreases the Maximum Log likelihood (MLL) \ significantly. Most of the reduction is between $n_{V}=3$ and $n_{V}=1$.
2. Case #4: Fitting $\alpha$ (in Cases #1 to #3, $\alpha=1)$ yielded an MLE that is very close to the assume value throughout the paper 0.92 (95%CI 0.74-1). When $\alpha=0,$ the MLL is -1986.579.
3. Case #5, we set $\omega=1$, while in Case #1-#4, it was 0.8 (reduced infectivity of BTI), the infection prevented by the third dose increases to .483 (95% CI 1.106;1.866) millions.
4. Case #6 is identical to Case #1 except for 10 nodes used in the transmission rate (evenly spanning over the study period). Although the MLL is much poor, the MLE of $\kappa$ and Infection prevented by the third dose are almost identical to Case #1.

These sensitivity analyses justified the choice of Case # 1 as the baseline model in the main text.

**Table A** Summary of Sensitivity Analysis

|  | MLL | $\omega$ | $n_{V}$ | $n_{\beta}$ | MLE and 95% for $\kappa$ or $\alpha$ | Infection prevented by the third dose (in millions) |
| --- | --- | --- | --- | --- | --- | --- |
| 1 | -1918.9 | 0.8 | 5 | 13 | 3.2 (2.9;3.633) | 1.219 (0.871;1.571) |
| 2 | -1926.9 | 0.8 | 3 | 13 | 2.867 (2.633;3.367) | 1.201 (0.83;1.586) |
| 3 | -1958.2 | 0.8 | 1 | 13 | 2.467 (2.233;2.6) | 0.748 (0.466;1.1) |
| 4 | -1918.9 | 0.8 | 5 | 13 | 0.92 (0.74;1) | 1.219 (0.871;1.571) |
| 5 | -1920.4 | 1 | 5 | 13 | 3.2 (2.9;3.667) | 1.483 (1.106;1.866) |
| 6 | -1962.7 | 0.8 | 5 | 10 | 3.2 (2.833;3.667) | 1.21 (0.791;1.604) |

In Case #3, we find that with an Exponentially distributed model ($n_{V}=1)$ the MLL is far worse than our baseline model. Thus we demonstrated that the duration of vaccine-induced immunity protection is better modelled as a peaked Gamma distribution (as in the baseline model) and having an initial period (the first 2-3 months) in which there is little immunity waning followed by a period of rapid waning.

**Figure C.** A comparison of an Exponential distribution and a Gamma distribution with the same mean.

A summary of assumed and estimated parameters is given in Table B. We estimate 17 parameters in our baseline model, including 13 parameters for the transmission rate, 4 extra parameters including $\rho_{1,2}$, $\kappa$, and $\tau$, besides initial conditions.

**Table B** Summary of assumed and estimated parameters

| Parameters | Symbol | Assumed parameter values /Intervals | Estimates (95%CI) |
| --- | --- | --- | --- |
| Initial susceptible | S | 0.95 of the popsize | 0.794 for <60, 0.156 for 60+ |
| Initial exposed | E | <0.001 of the popsize | 6.984287e-05 for <60, 6.984287e-06 for 60+ |
| Initial infectious | I | <0.001 of the popsize | Same as E |
| Initial recovered | R | ~0.05 of the popsize | 0.0416 for <60, 0.0082 for 60+ |
| Vaccinated class | $n_{V}$ | 5 |  |
| Population size | N | 9.29million |  |
| Population proportion of <60 |  | 0.836 |  |
| Transmission rate | $\beta(t)$ | [0,500] (13 nodes) | Before Dec 2020: 189.3, 67.1, 92.3, 155.5, 86.9, 141.8, 75.8, 90.6, 131.6; After Dec 2020: 122.9, 214.3, 276.6, 195.1, |
| Vaccination rate (second dose) | $\tilde{v}$ | From data |  |
| Vaccination rate (third dose) | $\tilde{b}$ | From data |  |
| Reduced susceptibility | $\varepsilon$ | 0.8 |  |
| Reduced infectivity | $\omega$ | 0.8 |  |
| Rate from latent to infectious | $\sigma$ | 1/2 per day |  |
| Recovery rate | $\gamma$ | 1/3 per day |  |
| Immunity types proportion | $\eta_{i}$, i=1,2,3. | $\eta_{1}=\eta_{3}=0.1$,  $\eta_{2}=0.8$. |  |
| Reporting ratio | $\rho_{i}$ | [0.4,1] | 44.57% for <60 and 50.2% for 60+ |
| Vaccine immunity waning rate | $\kappa$ | Estimated (per year) | 3.2 (2.9, 3.63) per year |
| Vaccine efficacy of the third dose | $\alpha$ | Estimated | 0.92 (0.74;1) |
| Over dispersion in reporting | $\tau$ |  | 0.159 |

The reporting process is as follows. We integrate $\rho_{i}\sigma E_{i}$ for a week to get simulated weekly reported cases $Y_{t}$, and assume

where $C_{t}$ is the corresponding reported cases and $\tau$ controls over dispersion in reporting.

**Section 4 : Stringency index**

The stringency index composes 9 response indicators to measure the strictness of the policies issued in response to the Covid-19 (6). The response indicators are: workplace closures, school closures, restrictions on public gatherings, cancellation of public events, stay-at-home requirements, closures of public transport, public information campaigns, international travel controls, and restrictions on internal movements. The index value is scaled from 0 to 100, and the higher score reveals the stricter policy response (6).

The calculation of stringency index is described in equation ,

$\text{stringency index}=\frac{1}{k}\sum_{j=1}^{k} I_{j}$,

where $k$ is the number of indicators and equals to 9 in this equation, and $I_{j}$ is the sub-score of each indicator (7). . The sub-score of the indicators are calculated by equation,

$I_{j,t}=100\times\frac{v_{j,t}-0.5(F_{j}-f_{j,t})}{N_{j}}$,

where $N_{j}$is the maximum value of indicator j, $F_{j}$ is the binary flag variable($F_{j}=1,0$), $v_{j,t}$ is the recorded policy value on given day t, and $f_{t,j}$ is the recorded binary flag. Some indicators such as school closures and workplace closures etc. are along with binary flag variable which equals to 1 or 0 (7). The flag variable is related to extra information like geographic scope of certain policy, sectoral scope of revenue support and the funded vaccination by government or individuals(7). Details of the indicator’s values and variables are shown in the codebook of Oxford Covid-19 Government Response Tracker (8). Fig D compares the stringency index and our estimated transmission rate, the MOH reproductive number, and the proportion of Delta variant. The estimated transmission rate is equivalent to a time-varying basic reproductive number, which explains the difference between it and the MOH Rt. The proportion of Delta drove the transmission rate to a high value, which is not fully reflected in the changes in the stringency index. Namely the relaxation of stringency and the invasion the Delta strain together increased the transmission rate in Summer 2021.

**Figure D**. A comparison of the stringency index (100-index)/5 with the estimated transmission rate (in unit of $\beta/\gamma$), and proportion of Delta variant sequenced. The peak of the transmission rate in July-September 2021 is likely a combined effect of the relaxing of stringency and invasion of the more transmissible variant. We downloaded stringency index for Israel from Our World in Data (9).

**Section 5: Vaccine efficacy of BNT162b2 in Qatar**

Our estimates are similar to those of Chemaitelly et al., derived from Qatar (10), which showed there was a sharp drop in VE around 4 months after the second dose (Fig E). This observation supports our assumption of a Gamma distributed waning time of VE. However, there are differences between the dynamics in those populations. In Israel, we set $\eta_{i}$=(0.1,0.8,0.1), and the shape parameter of the Gamma distribution at 5. If we set $\eta_{i}$=(0.0,0.7,0.3), and shape=5, our model output matches Chemaitelly et al 2021 well. Qatar had a Beta variant wave in May-June 2021, while Israel had an Alpha variant wave. It has been reported that the Beta variant has a stronger immune evasion ability than the Alpha variant (11), which could explain the observed difference in the VE drop.

**Figure E.** A comparison of Vaccine Efficacy (VE) waning functions. The blue vertical bars show VE in Qatar (10). The black vertical bars with circles show VE in Israel (12). Red and green curve show our VE model with different parameters. Both studies showed rapid drop of VE around four months after the second dose. This drop reflects a Gamma distributed duration (red curves) of vaccine induced protection, rather than an exponentially distributed (green curve).

**Section 6: Alternative scenarios**

We examined and analysed several alternative forms of the baseline model as sensitivity checks. First, we constructed a model that assumes the booster was never given (i.e., $\alpha=0$, see Table A in SI3). The model (lacking the effect of the booster) was then fitted to the full observed data set of the Israeli population that had experienced a booster. The failure of the model to fit the data well indicates that the actual booster had indeed modified the Israeli population’s epidemic dynamics. Without the third dose the model has a worse fit, decreasing the maximum log-likelihood (MLL) units by 30.9 units or a $\Delta AICc=61.8$ (the second order Akaike Information Criterion), which indicates a significant difference (13). We also calculated the profile of log-likelihood as a function of $\alpha$ (vaccine efficacy of the third dose), and found that the maximum likelihood estimate (MLE) of $\alpha$ is 0.96 with a 95% CI (0.72, 1). This justifies our choice of $\alpha=1$ in Fig 4.

We further fitted the baseline model, but with only a single stage in the *V* class, and thus an exponentially distributed duration of vaccination. In SI3 Table A, we show that under this assumption, the model fit is decreased by 21.3 log-likelihood units and a $\Delta AICc=42.5$ which is a significant difference. Thus, the Gamma distributed duration of vaccine-induced immunity protection employed in the baseline model is preferable to an exponentially distributed duration of protection.

We provide further sensitivity analysis on the number of stages in $V$ and the number of nodes used to vary the transmission rate in SI3 Table A. A summary of the predetermined and estimated parameters is provided in SI3 Table B. Our main conclusion holds when $n_{\beta}$ and $n_{V}$vary. We found that the third dose effects and the estimated $\kappa$ are consistent with the data, given the chosen model.

**References**

1. Israel urges vaccination for all teens, citing Delta variant.Reuters website.

2. Mallapaty S. Will COVID become a disease of the young? Nature. 2021;595(7867):343-4.

3. Regev-Yochay G, Amit S, Bergwerk M, Lipsitch M, Leshem E, Kahn R, et al. Decreased infectivity following BNT162b2 vaccination: a prospective cohort study in Israel. The Lancet Regional Health-Europe. 2021;7:100150.

4. Prunas O, Warren JL, Crawford FW, Gazit S, Patalon T, Weinberger DM, et al. Vaccination with BNT162b2 reduces transmission of SARS-CoV-2 to household contacts in Israel. Science. 2022:eabl4292.

5. Andrews N, Stowe J, Kirsebom F, Toffa S, Rickeard T, Gallagher E, et al. Covid-19 vaccine effectiveness against the Omicron (B. 1.1. 529) variant. New England Journal of Medicine. 2022;386(16):1532-46.

6. Hale T, Angrist N, Goldszmidt R, Kira B, Petherick A, Phillips T, et al. A global panel database of pandemic policies (Oxford COVID-19 Government Response Tracker). Nature Human Behaviour. 2021;5(4):529-38.

7. Toby Phillips HT. Methodology for Calculating Indices. 2021.

8. Toby Phillips HT. Codebook for the Oxford Covid-19 Government Response Tracker. 2021.

9. Hannah Ritchie EM, Lucas Rodés-Guirao, Cameron Appel, Charlie Giattino, Esteban Ortiz-Ospina, Joe Hasell, Bobbie Macdonald, Diana Beltekian, Max Roser. Coronavirus Pandemic (COVID-19) 2020 [Available from: <https://ourworldindata.org/coronavirus>.

10. Chemaitelly H, Tang P, Hasan MR, AlMukdad S, Yassine HM, Benslimane FM, et al. Waning of BNT162b2 vaccine protection against SARS-CoV-2 infection in Qatar. New England Journal of Medicine. 2021;385(24):e83.

11. Yang W, Shaman J. Development of a model-inference system for estimating epidemiological characteristics of SARS-CoV-2 variants of concern. Nature Communications. 2021;12(1):1-9.

12. Goldberg Y, Mandel M, Bar-On YM, Bodenheimer O, Freedman L, Haas EJ, et al. Waning immunity after the BNT162b2 vaccine in Israel. New England Journal of Medicine. 2021.

13. Anderson D, Burnham K. Model selection and multi-model inference. Second NY: Springer-Verlag. 2004;63(2020):10.
